# Individual body size as a predictor of lipid storage in Baltic Sea zooplankton

**DOI:** 10.1093/plankt/fbz010

**Published:** 2019-03-30

**Authors:** Elena Gorokhova

**Affiliations:** Department of Environmental Science and Analytical Chemistry, Stockholm University, SE-10691 Stockholm, Sweden

**Keywords:** neutral to polar lipid ratio, body size, Baltic zooplankton, individual protein content

## Abstract

The size structure of a zooplankton community is frequently used as a trait reflecting functional properties, including biochemical composition. Therefore, a shift in zooplankton body size can reflect shifts in the nutritional quality of zooplankton. In dominant Baltic copepods and cladocerans, neutral to polar lipid ratio (NL/PL ratio), a proxy for the mass-normalized lipid storage, was determined and related to individual body weight. A significant relationship between the NL/PL ratio and body weight was found; the latter was the strongest and the most significant predictor of the lipid storage capacity across different species and developmental stages. These findings provide support for using mean body weight in zooplankton community as a proxy for lipid storage capacity of zooplankton prey and justify applicability of zooplankton mean size as an indicator of nutritional conditions for Baltic zooplanktivores.

## INTRODUCTION

In aquatic systems worldwide, changes in stocks and composition of zooplankton induced by climate change and fisheries pressures have been observed (Heath, 2005). These alterations in zooplankton communities may cause cascading effects and contribute to ecosystem regime shifts (Alheit *et al.*, 2005; Daskalov *et al.*, 2007) In accordance with the “food-quality-limitation hypothesis” (Litzow *et al.*, 2006), a climate-induced shift in prey for pelagic fish has apparently occurred in the Baltic Sea. Changes in temperature and salinity were associated with changes in both abundance and composition of zooplankton communities (Möllmann *et al.*, 2008), with cascading effects on fish growth (Möllmann *et al.*, 2005), body condition (Flinkman *et al.*, 1998) and recruitment (Köster *et al.*, 2005).

Prey-mediated effects on fish may have at least two bottom-up mechanisms: food availability and its quality. Baltic herring are visual predators and selective planktivores, preferring large-sized conspicious zooplankton (Lazzaro, 1987), such as older stages of calanoid copepods and ovigerous females of copepods and cladocerans (Flinkman *et al.*, 1992). Declines in abundances of large-bodied zooplankters associate with changes in salinity and stratification have been implicated in the deterioration of fish growth conditions, indicated by decreased weight-at-age (WAA) and fat reserves in fish (Flinkman *et al.*, 1998). The qualitative aspects are much less understood but have been linked to the taxonomic composition of the prey, with large copepods considered the most valuable due to their high lipid content (Kattner *et al.*, 2007, 1981).

The size structure of a zooplankton community is frequently used as a proxy for functional properties, because many ecological characteristics are relatable to this trait, e.g. growth and metabolic rates, prey size range (Peters, 1983) and predator preference for prey (Scharf *et al.*, 2000). Hence, a shift in zooplankton body size can dramatically affect main ecosystem properties, including feeding conditions for fish (Peters, 1983). In line with this, mean zooplankter size has been suggested as a possible indicator of fish feeding conditions in the Baltic Sea (Gorokhova *et al.*, 2016). Moreover, for several Baltic areas, a significant decrease in average mean body size in zooplankton community over the last decade(s) have been found in several areas: the Gulf of Finland, Baltic proper, Bornholm Basin (Gorokhova *et al.*, 2016) and the Gulf of Riga (Simm *et al.*, 2014). However, some caution is necessary when inferring food quality from body size, because this relationship can be affected by community composition, ontogenetic changes within populations and feeding ecology of species involved.

Body size and nutritional quality of zooplankton have been associated with the storage of lipids resulting from a combination of phylogenetic, developmental and environmental components. Lipids contain high amounts of energy (~39 kJ g^–1^), thus representing a good proxy of physiological condition. The analysis of spatial variations in individual lipid content has provided insights into spatial differences in nutritional status of zooplankters, their feeding history, but also their quality as prey for fish (Lee *et al.*, 2006). When interpreting lipid content, it is important to differentiate storage from structural lipids. Neutral lipids are energy reserves for zooplankters (Lee *et al.*, 2006), and quantifying an individual’s neutral lipid reserves can indicate its nutritional status. Polar lipids provide the basic membrane matrix into which other constituents, such as proteins, are embedded (Vance and Vance, 2008) and, thus, they do not vary extensively with the nutritional condition but with the number of cells in the body (Lee *et al.*, 2006; Bader *et al.*, 2016). Since the quantity of the neutral lipids represents energy storage and quantity of polar lipids represents body size of an individual, the ratio of neutral to polar lipids (NL/PL ratio) in an animal can be used as an index of energy storage and nutritional condition scaled to body mass of the individual (Hentschel, 1998).

Here, using staining with Nile Red, we determined the NL/PL ratio, hereafter referred to as lipid content, in dominant Baltic Sea zooplankters of various life stages collected across broad areas. The primary questions addressed were: (i) Is there a relationship between lipid content and body size for Baltic zooplankton and (ii) Whether the strength of the lipid content/body size relationship changes across the main zooplankton groups.

## MATERIALS AND METHODS

### Zooplankton collection for biochemical analyses and sample preparation

In coastal and open areas of the northern Baltic Proper and the Bothnian Sea, zooplankton samples were collected on several occasions in 2007–2011 (Table I) using a WP2 net (mesh size 90 or 100 μm). Upon collection, zooplankton were rinsed with 0.2-μm filtered sea water (FSW) and sequentially filtered through different sieves (500, 250 and 35 μm) to get a rough size separation. The samples were then snap-frozen (−80°C) on a mesh, transported to the laboratory on dry ice, and stored for up to nine weeks. When preparing animals for the analysis, mesozooplankton samples were thawed and sorted on ice using a dissecting microscope (Wild Heerbrugg, ×50). The sorting was based on taxa, life stages (for the copepods and *Cercopagis pengoi*), and size classes (*Eubosmina coregoni maritima*); see Table II for the number of samples for each taxa and stage/size. To avoid a bias due to lipids allocated to egg mass (Lawrence, 1976), only females with invisible or only faintly visible gonad mass were used for the biochemical analyses.

**Table I:** Summary of zooplankton samples used for the biochemical analyses, collection sites, and dates. Species abbreviations: *Acartia bifilosa* (Ab), *Eurytemora affinis* (Ea), *Limnocalanus macrurus* (Lm), *Pseudocalanus acuspes* (Pa), *Centropages hamatus* (Ch), *Temora longicornis* (Tl), *Eubosmina coregoni maritima* (Em), and *Cercopagis pengoi* (Cp).

Station	Location, area	Geographic coordinates and depth	Month, Year	Number of samples per species							
				Ab	Ea	Lm	Pa	Ch	Tl	Em	Cp
H4	Himmerfjärden Bay, Northern Baltic Proper	N 58°59'02, E 17°43'50; 30 m	Jun-2007	6	12					12	3
B1	Askö station, Northern Baltic Proper	N 58°48'18, E 17°37'52; 40 m	Jul-2007	14	13		6	3	3	6	12
			Jul-2008		3		7	9		7	3
F62	Northern Baltic Proper	N 59°20.01’ E 23°15.81’ 95 m	Aug-2011	10	3	6	6	12	3	6	12
F64	Åland Sea	N 60°11.34’ E 19°08.55’ 285 m	Sep-2009	3	9	6	3	6		6	
US5b	Bothnian Sea	N 62°35.17’ E 19°58.13’ 214 m	Aug-2006	6	13	9	8			2	

**Table II:** Number of samples (all stations combined) and number of animals per sample (in parentheses) for each species and stage. Copepoda stages are: nauplii (N), copepodite stages I to III (CI-III), copepodite stages IV to V (CIV-V), and adults (males, M, and females, F).

Species	Stages					
	N	CI-III	CIV-V	M	F	*n*
Copepoda						
*Acartia bifilosa*	9 (20–25)	8 (15–22)	6 (12–23)	6 (10–15)	8 (10–15)	37
*Eurytemora affinis*	9 (23–35)	13 (16–25)	9 (13–19)	9 (10–12)	13 (10–12)	53
*Limnocalanus macrurus*		3 (8–10)	9 (5–10)	3 (6–15)	6 (4–5)	21
*Pseudocalanus acuspes*	6 (20–25)	15 (10–15)	9 (10–25)			30
*Centropages hamatus*	6 (20–24)	6 (12–15)	6 (10–13)	6 (3–10)	6 (6–12)	30
*Temora longicornis*	3 (20–25)	3 (20–23)				6
Cladocera	<0.3	0.3–0.4	0.4–0.5	0.5–0.7	>0.7	
*Eubosmina coregoni maritima*	6 (28–35)	10 (20–25)	9 (20–25)	11 (10–15)	3 (6–10)	39
	BSI	BSII/F	BSII-III/M	BSIII/PF	BSIII/GF	
*Cercopagis penoi*	3 (10–12)	6 (10)	6 (5)	9 (2–4)	6 (2–3)	30

For cladocerans, size classes (body length, mm) are used for *Eubosmina*, and barb stages (BS, I to III) and sex are used for *Cercopagis*, where BSI is barb stage I, sex is undifferentiated; BSII/F is females in barb stage II; BSII-III/M is males in barb stage II or III; BSIII/PF is parthenogenic females in barb stage III; and BSIII/GF is gametogenic females in barb stage III. Total number of samples per species is denoted as *n*.

These samples were compiled to include zooplankton species and groups varying in size, both intra- and interspecifically (Table II); in total, 240 samples were analyzed. Each sample was used to measure lipid to derive NL/PL ratio and protein to determine the individual protein content (μg ind.^−1^). The latter was used as a proxy for individual body size. To confirm the validity of this proxy, we examined the relationship between the measured protein content and species- and stage-specific biomass values (Hernroth, 1985); for *Cercopagis pengoi* (Onychopoda), which is not included in this list, we used mean stage-specific individual weights (Uitto *et al.*, 1999). In preparation for these measurements, sorted individuals were homogenized with 300 μL of distilled water for 2 min at 4°C using FastPrep® System (MP Biomedicals), sonicated at 50 Hz for 3 min, vortexed, and two 100-μL duplicate samples were taken for lipid analysis. After that, the rest of the sample was centrifuged at 2000 rpm for 7 min. The pellet was discarded, and the supernatant was used to generate two duplicate samples, 25 μL each, designated for protein analysis (Fig. S1, Supplementary Information).

### Lipid and protein analyses

Neutral and polar lipids were measured using staining technique with Nile Red (Enzo-Scientific, USA). A working solution of 50% DMSO (Sigma-Aldrich), 50% acetone, and 5 μg mL^−1^ Nile Red were prepared daily, and 100 μL were added to each well containing 100 μl homogenate. Blanks contained 100 μL of distilled water and 100 μL of the working solution. The plate was incubated in the dark with shaking at 37°C and fluorescence was read after 10 min. Lipid ratios were quantified using a FLUOstar Optima microplate reader (Labvision, Germany) in black solid flat-bottom microplates (Greiner Bio-One GmbH) at excitation/emission wavelengths of 485/520–560 nm for neutral lipids and 544/612 nm for polar lipids (Hentschel, 1998); 10 measurements well^−1^ at 0.2 s well^−1^ were taken for each analytical replicate (Supplementary Information, Fig. S1).

Protein concentration in the homogenates was assayed using a modification of the Lowry Protein Assay as the Micro BCA Protein Assay Kit (Pierce) with a linear working range of 2–50 μg mL^−1^. The analysis was done in a microplate format, where 50 μL (for nauplii and smallest *Eubosmina*) or 25 μL (for all other zooplankters) of the homogenate per well were mixed with 50 or 75 μL of distilled water. To each well, 100 μL of the working reagent containing bicinchoninic acid (BCA) were added; the plate was incubated at 37°C for 2 hours and the absorbance was read at 560 nm. Bovine serum albumin was used as a standard at five dilutions on each plate. The protein concentration and the number of animals per sample (Table II) were used to estimate individual protein mass (μg ind.^−1^).

### Data analysis and statistics

Generalized linear models (GLM) with normal error structure and log link as implemented in STATISTICA 8.0 (StatSoft, 1984–2007) were used to evaluate (i) relationships between individual protein mass (*Protein*) and body wet weight (*WW*) and (ii) effects of *Protein*, *Species* and *Species* × *Protein* interaction term on the NL/PL ratio in zooplankton. The data were Box-Cox transformed to improve normality of the model residuals. The post-hoc Newman–Keuls test was used for zooplankton species grouping based on their observed NL/PL ratio. Also, using the transformed data, adjusted means for NL/PL ratio in zooplankton species were compared by ANOVA with Tukey’s multiple comparisons.

## RESULTS

**Fig. 1.**
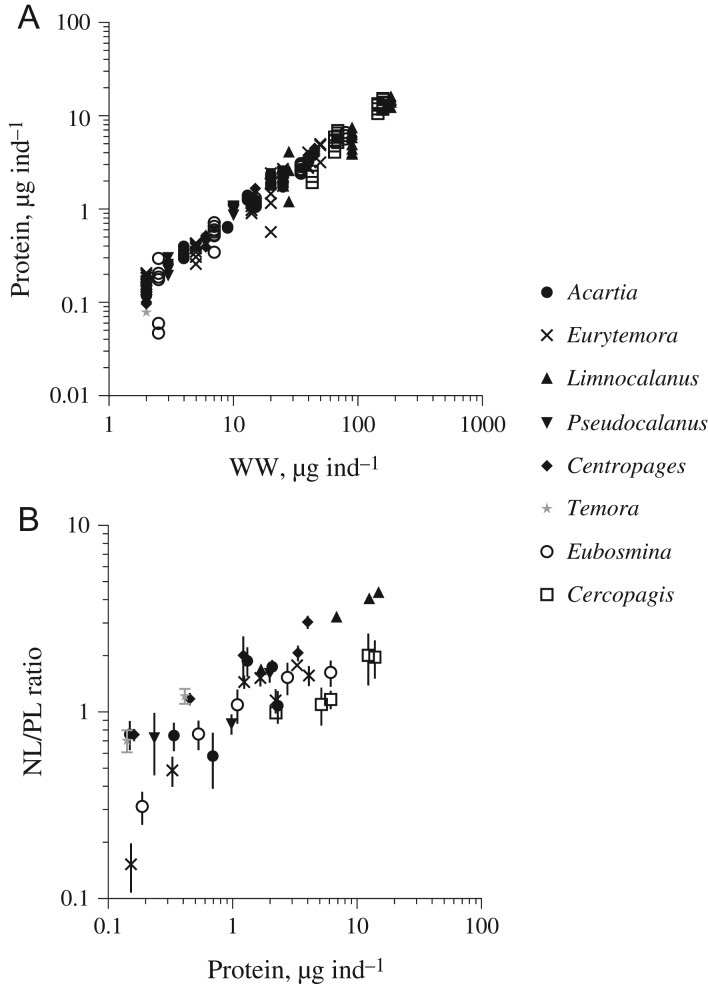
Relationships between (**A**) individual protein mass (Protein, μg ind.^−1^) and wet weight (WW, μg ind.^−1^). As WW, the species- and stage-based individual weights were used (Hernroth, 1985); and (**B**) species- and stage-specific NL/PL ratio (mean ± SD) and individual protein content. See Table II for species and stages used for the analyses as well as the number of samples analyzed. Observe that log-log scale was used for both relationships.

In all species tested, individual protein mass increased with body mass in a linear fashion with *R*^2^ > 0.9 (Fig. 1A; Supplementary Information, Fig. S2). The overall regression over the mass range of 2 to ~200 μg WW ind.^−1^ was highly significant, with WW explaining 96% of the variance in the individual protein mass. This relationship indicates relatively stable contribution of protein to the biochemical composition of the tested microcrustaceans across species and stages. Thus, both protein mass and wet weight can be used as metrics for zooplankton body size.

In both cladocerans and copepods, the NL/PL ratio varied among the species and stages (Supplementary Information, Fig. S3). In copepods, within-stage variability was significant for females of *E. affinis* (one-way ANOVA: *F*_2,6_ = 112.4; *P* < 0.0001; *R*^2^ = 0.96) and marginally significant for nauplii of *A. bifilosa* (*F*_2,6_ = 5.043; *P* = 0.052; *R*^2^ = 0.63) and older copepodites of *P. acuspes* (*F*_2,6_ = 4.258; *P* = 0.071; *R*^2^ = 0.58) collected at different stations. In cladocerans, significant between-station differences were observed for the larger (0.5–0.7 mm) individuals of *Eubosmina*: (*F*_3,7_ = 4.421; *P* = 0.048; *R*^2^ = 0.65). No other samples within the same species/stages or size classes differed significantly among the sampling stations.

**Fig. 2.**
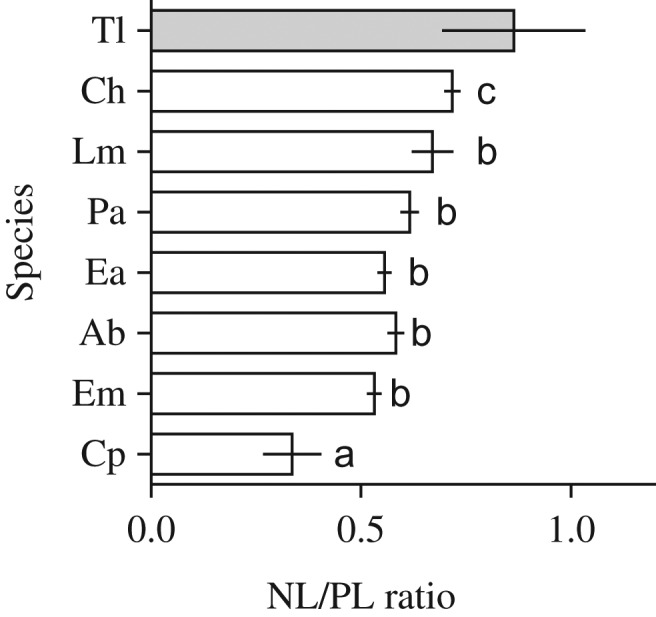
Adjusted means (±SE; Box-Cox transformed values) for NL/PL ratio in zooplankton species using size as a covariable (GLM analysis; Table IIIA). The means were compared by ANOVA with Tukey’s multiple comparisons test using all species (*n* varied 21 to 47 among the species), excluding *Temora longicornis* (Tl, gray bar) for which only six observations skewed to the early developmental stages (mostly nauplii and earlier copepodites) were available. Non-matching letters indicate significant differences between the species. Note that between-species similarity was different when using unadjusted data (Table IIIB). See Table I for species abbreviations (*Y*-axis) and Fig. S2 (Supplementary Information) for the non-transformed values for each species and station.

Across the species and stages, body size was a strong predictor for size-specific lipid reserves. The NL/PL ratio significantly increased with body size assayed as protein mass in a non-linear, yet monotonic fashion; moreover there was a significant *Species* effect, but not a *Species* × *Size* interaction (Table IIIA). The latter indicated that the relative increase in NL/PL driven by size is uniform for all species tested. The model described 76% of the variance in the NL/PL ratio and was highly significant. Moreover, the effect size of *Species* was only 0.08, compared to the cumulative value of 0.64 contributed by both the body size effect and the centered intercept reflecting the estimated NL/PL ratio at the average size (Table IIIA). When the mean values were compared among the species without considering differences in their body size, there were three statistically homogenous groups reflecting low, medium and high levels of NL/PL ratio (Table IIIB). However, when the adjusted means of the NL/PL ratio that reflect size-specific capacity to accumulate storage lipids were compared, the grouping was different, with *C. hamatus* (and, perhaps, *T. longicornis*) and *C. pengoi* having the maximal and minimal size-specific lipid storage (Fig. 2). For *C. pengoi*, the differences between the unadjusted (Table IIIB) and adjusted (Fig. 2) values were related to the largest body size resulting in the lowest size-specific NL/PL values.

**Table III:** Statistical summary of testing differences in NL/PL ration among the species: (A) general linear model (GLM) examining effects of body *Size* measured as individual protein content (μg ind^−1^), *Species* and *Species × Size* interaction term on the NL/PL ratio in zooplankton; and (B) post-hoc Newman–Keuls test for species grouping based on their observed NL/PL ratio; Homogenous Groups, *α* = 0.05; Error: Between MSE = 0.0098, df = 225. Total number of observations is 171 and 69 for copepods (6 species) and cladocerans (2 species), respectively (Fig. 1B).

(A)								
Predictors	SS	df	MS	F	*p*-value	ηp^2^	Non-centrality	Power
Intercept	1.156	1	1.156	102.9	**<0.0000**	0.313	102.9	1.000
Species	0.237	7	0.033	3.025	**0.005**	0.086	21.17	0.934
Size	1.208	1	1.208	107.6	**<0.0000**	0.323	107.6	1.000
Species × size	0.061	7	0.008	0.7793	0.605	0.023	5.455	0.332
Error	2.527	225	0.011					

(B)				
Species	Mean	Low NL/PL	Medium NL/PL	High NL/PL
*E. c. maritima*	0.48	×		
*E. affinis*	0.49	×		
*T. longicornis*	0.49	×		
*A. bifilosa*	0.51	×		
*P. acuspes*	0.56	×		
*C. pengoi*	0.65		×	
*C. hamatus*	0.68		×	
*L. macrurus*	0.93			×

Effect size in GLM was determined using partial eta-squared (ηp^2^); adjusted *R*^2^ for the model is 0.76, *F* value is 52.62, *p* < 0.0001. Significant effects are in bold.

## DISCUSSION

Body size is a master trait, with allometric implications not only for physiological rates but also for a biochemical composition (Guisande, 2006; Ikeda and McKinnon, 2012). Allometric relationships involving functional and biochemical traits facilitate analysis of adaptations to the abiotic and biotic environments, such as trade-offs among different functions within an organism as well as consequences for adjacent trophic levels in the food web. However, there is a paucity of data on the proportional biochemical composition of marine and freshwater zooplankton with respect to specific protein and lipid content (Hébert *et al.*, 2016). We found that the NL/PL ratio has significant within-stage variability in certain zooplankton species related to the sampling site and the causes of this variability are unclear, because these samples were collected at different months and years (Supplementary Information, Fig. S3). When the protein content was used as a substitute for stage or size class, the overall relationship between the NL/PL ratio and protein content was significant, with 76% of the variance explained. Therefore, in crustacean zooplankton species commonly occurring in the Baltic Sea, body mass appears to be a useful proxy for protein content and size-specific lipid storage (Fig. 1A; Supplementary Information, Table S1).

In the analyzed set of species and ontogenetic stages, the NL/PL ratio varied ~8-fold (Fig. 1B). Zooplankton species that had greater mass-specific lipid stores were *Limnocalanus*, *Centropages* and older copepodites of *Pseudocalanus*. These taxa occur predominantly in the northern (*Limnocalanus, Pseudocalanus*) and southern (*Centropages, Pseudocalanus*) parts of the Baltic Sea. Although the biochemical composition of the lipids could not be evaluated from our data, it is most likely that all three species being of marine origin and inhabiting deeper and colder layers accumulate large quantities of wax esters (Clarke and Peck, 1991; Vanderploeg *et al.*, 1998). By contrast, species of freshwater origin, such as *Eurytemora affinis* and bosminids, accumulate mostly triacylglycerols as shown for other freshwater zooplankton (Cavaletto *et al.*, 1989). Therefore, in the Baltic zooplankton, the body size is indicative of both quantities of the neutral lipids and—probably—their composition. The only species with substantially lower lipid storage on the mass-specific basis was the invasive onychopod cladoceran *Cercopagis pengoi* (Fig. 2), also of freshwater origin (Mordukhai-Boltovskoi, 1965). Since its appearance in the Baltic Sea in the mid-1990-ties, *Cercopagis* has become a common prey for zooplanktivorous fish (Gorokhova *et al.*, 2004). Moreover, *Cercopagis* is positively selected by the Baltic herring (Gorokhova *et al.*, 2004; Livdāne *et al.*, 2016). One can speculate that relative availability of this relatively large (Fig. 1A), yet nutritiously poor—compared to the copepods of similar size—prey may contribute to the variability in body condition of herring and sprat in the Baltic Sea (Casini *et al.*, 2006).

Though having information on the fatty acid composition of zooplankton prey (Kattner *et al.*, 2007) as well as better taxonomic resolution of diet analysis (Robert *et al.*, 2014) are needed to understand the biochemical basis of trophic interactions, the application NL/PL ratio as a proxy for lipid storage is a viable option for assessing prey quality. In the Baltic Sea, the lipid content is not significantly different between similarly-sized bosminids and copepods (Figs 1B, and 2), i.e. taxa that often dominate clupeid diets (Flinkman *et al.*, 1992). Therefore, mean body size rather than other commonly used metrics of zooplankton community structure based on the contribution of different taxonomic groups (e.g. the percentage of copepods or Cladocera/Copepoda ratio) can be used to evaluate the nutritional value of zooplankton standing stock in the Baltic Sea. This is particularly useful in this system, where the biomass structure of zooplankton communities varies substantially north to south, with a higher copepod contribution (mostly *Limnocalanus macrurus*) in the Gulf of Bothnia and Southern Baltic compared to the Baltic Proper (Gorokhova *et al.*, 2016). In the northern Baltic Proper, the small-sized copepods (*Acartia* spp. and *Eurytemora affinis*) and cladocerans (bosminids and podonids) contribute most to the total zooplankton biomass (Johansson *et al.*, 2004) and, consequently, to the prey available for zooplanktivores. In the southern Baltic Proper, the contribution of these taxa is lower than in its northern part, although even here they comprise a substantial part of summer zooplankton (Schulz *et al.*, 2012).

The empirical evidence that body size in the Baltic Sea zooplankton is a significant predictor of the lipid storage capacity across different developmental stages in dominant copepods and cladocerans provides insights for possible mechanisms of the bottom-up effects on fish body condition. During the last three decades, herring fat content has significantly decreased in the Bothnian Bay, Northern Baltic proper and Bornholm basin (Nyberg *et al.*, 2015), in concert with the decreased zooplankton mean size (Gorokhova *et al.* 2016). One might speculate that the decreasing fat content in herring could be related to the decline in the prey lipid content and its nutritional quality. For example, in the Northern Baltic proper and Bornholm, the decrease in the mean body size during 1990–2010 (Gorokhova *et al.* 2016) would correspond to ~30% decrease in zooplankton lipid content, provided that the relationships between the zooplankton individual weight and protein and between protein and NL/PL ratio established in our study hold true on the long-term perspective. Therefore, the decreased body condition of herring in the Baltic Sea during the last decades could be, at least partly, caused by the inadequate prey quality. The trends in zooplankton mean size and fish body condition call for more studies investigating the mechanisms of the bottom-up effects as driving forces behind changes in zooplankton and fish stocks.

## Supplementary Material

Supplementary_Information_fbz010
