# Supplementary material for: Individual body size as a predictor of lipid storage in Baltic Sea zooplankton
Source: J Plankton Res. 2019 Mar 30;41(3):273–80. doi: 10.1093/plankt/fbz010 (PMC6821285; doi:10.1093/plankt/fbz010)
Supplement: Supplementary_Information_fbz010 [file supplementary_information_fbz010.docx]

**Supplementary Information**

**Individual body size as a predictor of lipid storage in Baltic Sea zooplankton**

Elena Gorokhova

Department of Environmental Science and Analytical Chemistry, Stockholm University, SE-10691 Stockholm, Sweden

**Table S1.** Linear regressions for protein mass (μg ind.^-1^) as a function of individual wet weight (μg ind.^-1^). The wet weight values are adopted from the recommendation for biomass assessment in zooplankton monitoring of the Baltic Sea (Hernroth 1985). Species and stages used for protein analysis are listed in Table 2; SE is standard error, *n* is number of observations. See Table S2 for the raw data.

| Regression parameters | 1. *bifilosa* | *E. affinis* | *L. macrurus* | *P. acuspes* | *C. hamatus* | *T. longicornis** | *E. c. maritima* | *C. pengoi* |
| --- | --- | --- | --- | --- | --- | --- | --- | --- |
| Slope ± SE | 0.098 ± 0.004 | 0.086 ± 0.004 | 0.082 ± 0.006 | 0.104 ± 0.003 | 0.091 ± 0.002 | 0.095 ± 0.007 | 0.077 ± 0.001 | 0.090 ± 0.004 |
| Intercept ± SE | -0.042 ± 0.061 | -0.038 ± 0.083 | -1.154 ± 0.738 | -0.052 ± 0.039 | -0.076 ± 0.067 | -0.073 ± 0.032 | 0.006 ± 0.040 | -0.711 ± 0.482 |
| R^2^ | 0.94 | 0.91 | 0.91 | 0.97 | 0.98 | 0.97 | 0.98 | 0.94 |
| P value | < 0.0001 | < 0.0001 | < 0.0001 | < 0.0001 | < 0.0001 | 0.0002 | < 0.0001 | < 0.0001 |
| *n* | 37 | 47 | 21 | 30 | 30 | 6 | 40 | 30 |

*Note that for *Temora longicornis* only 6 observations skewed to the early developmental stages (mostly nauplii and earlier copepodites) were available; therefore, the regression coefficient and the *p*-value should be treated with caution.

**Table S2.** Station-specific data on zooplankton body size (wet weight) and NL/PL ratio; n – number of samples analyzed.

| **Group** | **Species** | **Station** | **Wet weight,**  **µg ind^-1^** | **Mean NL/PL ratio** | **SD** | **n** |
| --- | --- | --- | --- | --- | --- | --- |
| copepods | Acartia | H4 | 2 | 0.76 | 0.27 | 3 |
| copepods | Acartia | H4 | 9 | 0.60 | 0.05 | 3 |
| copepods | Acartia | B1 | 2 | 0.69 | 0.03 | 3 |
| copepods | Acartia | B1 | 4 | 0.67 | 0.09 | 2 |
| copepods | Acartia | B1 | 13 | 1.62 | 0.07 | 3 |
| copepods | Acartia | B1 | 20 | 1.59 | 0.10 | 3 |
| copepods | Acartia | B1 | 25 | 1.13 | 0.12 | 3 |
| copepods | Acartia | F62 | 2 | 0.98 | 0.19 | 3 |
| copepods | Acartia | F62 | 13 | 1.58 | 0.19 | 3 |
| copepods | Acartia | F62 | 20 | 1.61 | 0.10 | 3 |
| copepods | Acartia | F64 | 25 | 1.06 | 0.09 | 2 |
| copepods | Acartia | US5b | 4 | 0.49 | 0.09 | 3 |
| copepods | Acartia | US5b | 25 | 1.16 | 0.13 | 3 |
| copepods | Eurytemora | H4 | 2 | 0.68 | 0.09 | 3 |
| copepods | Eurytemora | H4 | 5 | 0.50 | 0.15 | 3 |
| copepods | Eurytemora | H4 | 40 | 1.86 | 0.14 | 3 |
| copepods | Eurytemora | H4 | 50 | 1.47 | 0.33 | 3 |
| copepods | Eurytemora | B1 | 5 | 0.47 | 0.08 | 3 |
| copepods | Eurytemora | B1 | 14 | 1.65 | 0.15 | 3 |
| copepods | Eurytemora | B1 | 20 | 1.76 | 0.13 | 3 |
| copepods | Eurytemora | B1 | 25 | 1.02 | 0.16 | 4 |
| copepods | Eurytemora | B1 | 2 | 0.64 | 0.02 | 3 |
| copepods | Eurytemora | F62 | 20 | 1.70 | 0.15 | 3 |
| copepods | Eurytemora | F64 | 5 | 0.53 | 0.10 | 3 |
| copepods | Eurytemora | F64 | 14 | 1.34 | 0.18 | 3 |
| copepods | Eurytemora | F64 | 25 | 1.31 | 0.21 | 3 |
| copepods | Eurytemora | US5b | 2 | 0.73 | 0.01 | 3 |
| copepods | Eurytemora | US5b | 5 | 0.48 | 0.11 | 4 |
| copepods | Eurytemora | US5b | 14 | 1.53 | 0.10 | 3 |
| copepods | Eurytemora | US5b | 25 | 1.45 | 0.26 | 3 |
| copepods | Limnocalanus | F62 | 90 | 3.13 | 0.08 | 3 |
| copepods | Limnocalanus | F62 | 169 | 4.06 | 0.20 | 3 |
| copepods | Limnocalanus | F64 | 90 | 3.18 | 0.15 | 3 |
| copepods | Limnocalanus | F64 | 185 | 4.34 | 0.20 | 3 |
| copepods | Limnocalanus | US5b | 28 | 1.81 | 0.07 | 3 |
| copepods | Limnocalanus | US5b | 90 | 3.22 | 0.10 | 3 |
| copepods | Limnocalanus | US5b | 185 | 4.28 | 0.30 | 3 |
| copepods | Pseudocalanus | B1 | 3 | 0.68 | 0.09 | 3 |
| copepods | Pseudocalanus | B1 | 10 | 0.78 | 0.05 | 3 |
| **Table S2. Cont.** | |  |  |  |  |  |
| copepods | Pseudocalanus | B1 | 3 | 0.27 | 0.20 | 3 |
| copepods | Pseudocalanus | B1 | 10 | 0.78 | 0.08 | 4 |
| copepods | Pseudocalanus | F62 | 10 | 0.97 | 0.05 | 3 |
| copepods | Pseudocalanus | F62 | 20 | 1.58 | 0.19 | 3 |
| copepods | Pseudocalanus | F64 | 20 | 1.71 | 0.15 | 3 |
| copepods | Pseudocalanus | US5b | 10 | 0.86 | 0.13 | 5 |
| copepods | Pseudocalanus | US5b | 20 | 1.81 | 0.06 | 3 |
| copepods | Centropages | B1 | 6 | 1.30 | 0.07 | 3 |
| copepods | Centropages | B1 | 15 | 2.18 | 0.38 | 3 |
| copepods | Centropages | B1 | 40 | 2.20 | 0.35 | 3 |
| copepods | Centropages | B1 | 45 | 3.22 | 0.03 | 3 |
| copepods | Centropages | F62 | 2 | 0.88 | 0.23 | 3 |
| copepods | Centropages | F62 | 15 | 2.01 | 0.69 | 3 |
| copepods | Centropages | F62 | 40 | 2.06 | 0.05 | 3 |
| copepods | Centropages | F62 | 45 | 3.12 | 0.10 | 3 |
| copepods | Centropages | F64 | 2 | 0.60 | 0.07 | 3 |
| copepods | Centropages | F64 | 6 | 1.21 | 0.11 | 3 |
| cladocerans | Bosmina | H4 | 2.5 | 0.28 | 0.03 | 3 |
| cladocerans | Bosmina | H4 | 7 | 0.79 | 0.11 | 3 |
| cladocerans | Bosmina | H4 | 15 | 1.13 | 0.18 | 3 |
| cladocerans | Bosmina | H4 | 35 | 1.32 | 0.09 | 3 |
| cladocerans | Bosmina | B1 | 15 | 1.18 | 0.15 | 3 |
| cladocerans | Bosmina | B1 | 35 | 1.31 | 0.08 | 3 |
| cladocerans | Bosmina | B1 | 2.5 | 0.30 | 0.03 | 3 |
| cladocerans | Bosmina | B1 | 7 | 0.69 | 0.03 | 4 |
| cladocerans | Bosmina | F62 | 35 | 1.13 | 0.10 | 3 |
| cladocerans | Bosmina | F62 | 80 | 1.08 | 0.12 | 3 |
| cladocerans | Bosmina | F64 | 7 | 0.65 | 0.04 | 3 |
| cladocerans | Bosmina | F64 | 15 | 1.12 | 0.13 | 3 |
| cladocerans | Bosmina | US5b | 35 | 1.25 | 0.07 | 2 |
| cladocerans | Cercopagis | H4 | 159 | 1.65 | 0.31 | 3 |
| cladocerans | Cercopagis | B1 | 43 | 1.03 | 0.17 | 3 |
| cladocerans | Cercopagis | B1 | 69 | 1.08 | 0.35 | 3 |
| cladocerans | Cercopagis | B1 | 159 | 1.64 | 0.05 | 3 |
| cladocerans | Cercopagis | B1 | 145 | 1.66 | 0.20 | 3 |
| cladocerans | Cercopagis | B1 | 65 | 0.96 | 0.06 | 3 |
| cladocerans | Cercopagis | F62 | 65 | 1.12 | 0.09 | 3 |
| cladocerans | Cercopagis | F62 | 69 | 1.30 | 0.18 | 3 |
| cladocerans | Cercopagis | F62 | 159 | 1.57 | 0.08 | 3 |
| cladocerans | Cercopagis | F62 | 145 | 1.63 | 0.32 | 3 |

**Figure S1.** Workflow for analysis of the NL/PL ratio and protein content in zooplankton. Stars indicate data collection points.


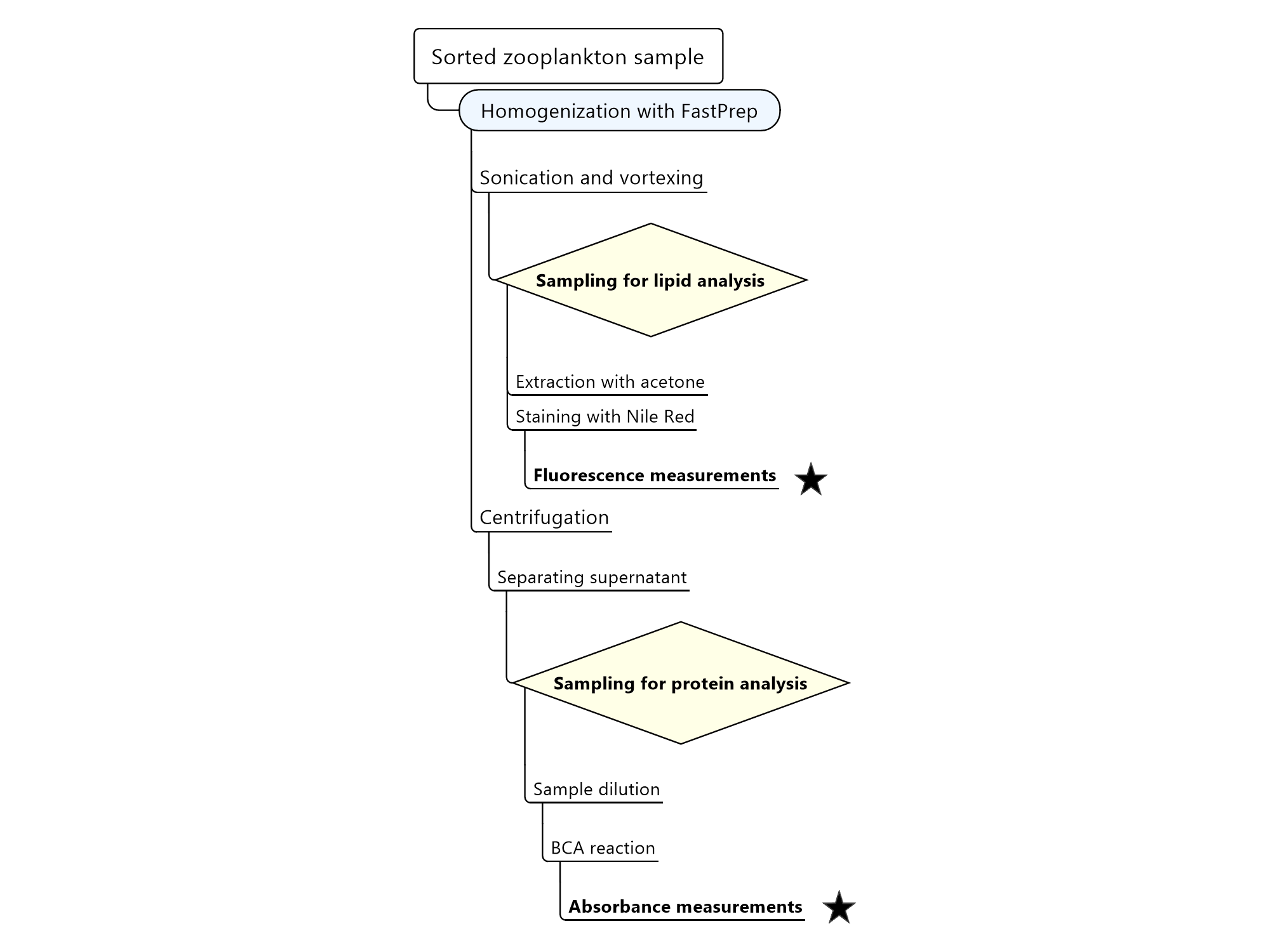


**Figure S2.** Protein mass (μg ind.^-1^) to wet weight (WW; μg ind.^-1^) regressions for Baltic zooplankters. Each data point is a sample composed by individuals of the same species and stage/size group; see Table 2 for the list of stages and number of individuals per sample. See also Table S1 for the regression details.

**Figure S3**. Stage-specific NL/PL ratio in the calanoid copepods and cladocerans collected in the coastal areas of the north-western Baltic proper (stations H4 and B1), north-eastern Baltic proper (F62), Åland Sea (F64) and offshore area of the Bothnian Sea (US5b) in 2006-2011.
